# Surface-enhanced Raman scattering used to study the structure of layers formed on metal surfaces from single-stranded DNA and 6-mercaptohexan-1-ol: influence of hybridization with the complementary DNA and influence of the metal substrate†

**DOI:** 10.1039/d2ra05318g

**Published:** 2022-12-08

**Authors:** Aleksandra Michałowska, Aleksandra Gajda, Agata Kowalczyk, Jan L. Weyher, Anna M. Nowicka, Andrzej Kudelski

**Affiliations:** Faculty of Chemistry, University of Warsaw Pasteura 1 Str. PL 02-093 Warsaw Poland akudel@chem.uw.edu.pl; Institute of High Pressure Physics of the Polish Academy of Science Sokolowska 29/37 Str. PL 01-142 Warsaw Poland

## Abstract

Capture single-stranded DNA with an attached alkanethiol linking moiety (capture HS-ssDNA) and 6-mercaptohexan-1-ol were chemisorbed on nanostructured GaN covered with sputtered layers of plasmonic metals (like silver and gold). The structure of the formed layer was determined by surface-enhanced Raman scattering (SERS) measurements. Hybridization with the target ssDNA, complementary to the chains of immobilized capture HS-ssDNA, induced changes in the conformation of the chains of chemisorbed ω-substituted alkanetiols (6-mercaptohexan-1-ol and the alkanethiol linking moiety of HS-ssDNA). Such changes are significantly larger in the case of experiments on silver than on gold and gold/silver SERS substrates. This means that silver substrates are significantly more promising for the SERS observation of such hybridization-induced rearrangements than the gold substrates previously used. Although the sputtered metal films have a nanograin structure, the nanostructuring of the GaN substrates plays an important role in the SERS-activity of this nanomaterial.

## Abstract 2

Hybridization-induced rearrangement of alkanethiolate chain forming linking moiety of ssDNA and a blocking layer is significantly larger for systems formed on silver substrates than on previously used gold surfaces.

## Graphical abstract

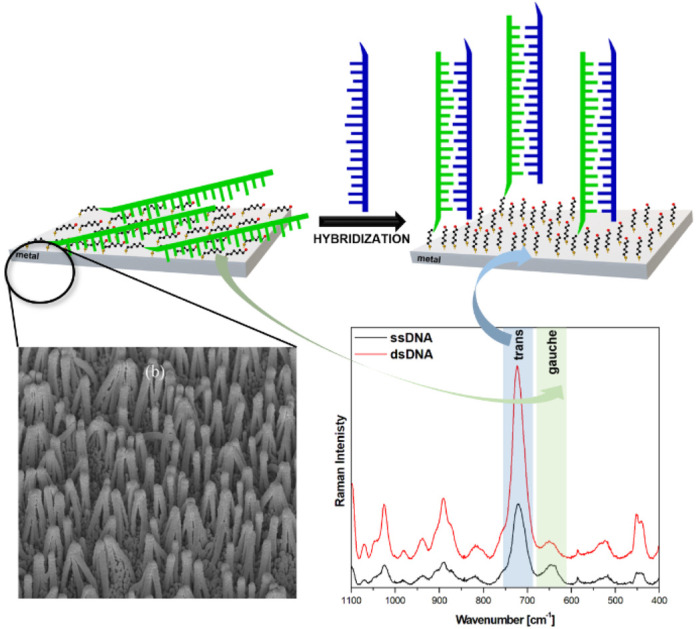

## 1. Introduction

There is strong interest in developing new methods for the sensitive identification of specific DNA fragments – such as circulating free tumour DNA – in samples of blood and certain other medical materials. Identifying specific DNA (as mentioned above, circulating tumour DNA) can serve to detect mutations in genes of predictive and prognostic importance for molecularly targeted therapies, and can therefore influence clinical decisions.^1^ Currently, genetic predispositions for cancers are mainly studied using polymerase chain reaction (PCR) and next-generation sequencing (NGS).^2^ In some cases, however, the cost or waiting time of such methods are unsatisfactory, and so new methods of DNA analysis continue to be developed.

Among the ‘new’ analytical techniques that many groups are trying to use in the ultrasensitive identification of specific DNA fragments, a prominent place is held by surface-enhanced Raman scattering (SERS).^3–5^ There are many different approaches to applying SERS for the detection of DNA of a specific sequence (usually containing a mutation). In standard SERS DNA sensors, a so-called Raman reporter (a moiety having an exceptionally large cross section for Raman scattering)^6^ is used. In one group of the standard SERS DNA detection methods, the detection is based on the rearrangement of the DNA chain leading to a Raman reporter being placed close to the plasmonic nanostructure, or a Raman reporter being removed from the proximity of the plasmonic structure when a DNA fragment with a given sentence is present in the analyzed sample.^7–9^ This ‘movement’ of the Raman reporter is effected by DNA hybridization leading to the formation of double-stranded DNA or to the formation or decomposition of a hairpin chain (known as a ‘stem-loop’ configuration). Because the efficiency of the generation of SERS signal strongly decreases with increasing distance from the plasmonic nanostructure (roughly as a function of *r*^−10^),^10–12^ the measured SERS signal is very strong when the Raman reporter is close to the SERS-active plasmonic nanostructure, and weak when it is farther away.

Another approach often applied in standard DNA SERS sensors utilizes the agglomeration of various nanoparticles induced by the hybridization of complementary fragments of DNA (fragments of DNA are immobilized on the nanoparticles on which agglomeration is observed). Where only plasmonic nanoparticles agglomerate, the agglomeration is detected by observing the very large increase in the intensity of the recorded SERS signal of a Raman reporter immobilized on the surface of the agglomerated nanoparticles^13–15^ (due to plasmonic coupling, the strongest electric field enhancement is generated in the slits between the plasmonic nanostructures, and therefore, the agglomeration of the plasmonic nanostructures strongly increases their SERS activity^5,10^). The agglomeration induced by the presence of specific DNA fragments can be also achieved in a similar manner for a mixture of magnetic and plasmonic nanoparticles.^16–18^ Here, hybridization (and hence, the presence of the target DNA) is manifest in the appearance of the possibility of a rapid concentration of the SERS nanomaterial in the magnetic field.

**Fig. 1:**
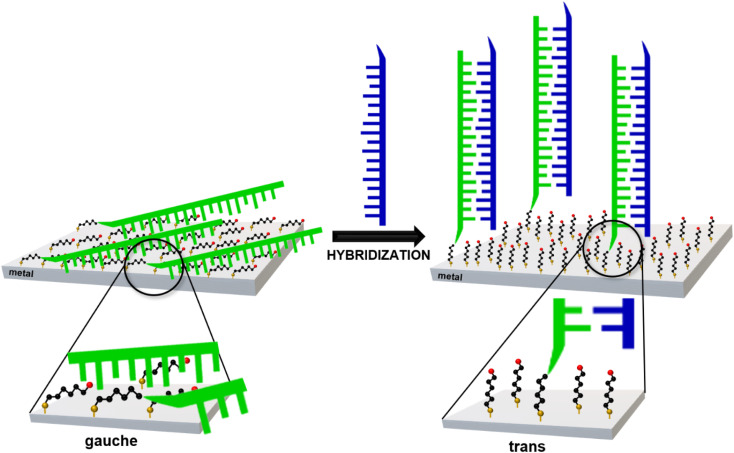
Scheme of the rearrangement of the chemisorbed 6-mercaptohexan-1-ol and the alkanethiolate linking moiety caused by DNA hybridization.

In 2019, Kowalczyk *et al.* proposed a new strategy for identifying gene mutations using SERS spectroscopy.^19^ It was found that, when one immobilizes ‘thiolated’ (with an attached alkanethiol moiety) capture single-stranded DNA (HS-ssDNA) and 6-mercaptohexan-1-ol on a SERS-active gold surface, and the structure is incubated with a sample containing DNA complementary to the immobilized capture HS-ssDNA, the presence of the target ssDNA induces hybridization, which causes a change in the conformation of the chains of chemisorbed ω-substituted alkanetiols (6-mercaptohexan-1-ol and the alkanethiol linking moiety *via* which the captured single-stranded DNA is attached to the gold surface) – see Fig. 1.^19^ That change is indicated by a characteristic change in the measured SERS spectrum (*i.e.*, due to the above-mentioned highly surface-sensitive mechanism of the SERS enhancement, the SERS spectra measured for such samples are dominated by the vibrations localized in the linking layer). In brief: the intensity ratio of the *ν*(C–S) bands of the *trans* and *gauche* conformer of the Au–S–C–C chain (alkane chains of both: chemisorbed 6-mercaptohexan-1-ol and the alkanethiol linking moiety *via* which the captured single-stranded DNA is attached to the gold surface) for samples that do not contain complementary DNA was slightly below 1, whereas this ratio was between *ca.* 1.1 and *ca.* 1.5 for the samples containing complementary DNA^19^ (due to the hybridization-induced rearrangement of the structure of the chemisorbed 6-mercaptohexan-1-ol and the alkanethiolate linking moiety, as shown in Fig. 1).

Previous experiments with monolayers formed from various non-substituted and ω-substituted alkanethiols have shown that the structure of the monolayer formed strongly depends on the kind of metal from which the substrate was made (in general, the orientation of the alkane chains is more perpendicular to the metal surface for layers formed on silver than on gold).^20,21^ Therefore, one might expect that the difference in the structure of mixed layers formed from ω-substituted alkanethiols (6-mercaptohexan-1-ol) and alkanethiols with an attached single-stranded (before hybridization) and double-stranded (after hybridization) DNA chain should also depend on the kind of plasmonic substrate used. In this work, we decided to investigate on which of the three standard plasmonic metals (gold, silver and copper) there would be the largest difference in the structure of the linking/blocking alkanethiol chains before and after hybridization of the capture HS-ssDNA with the target ssDNA. Such information can enhance our knowledge about the properties of certain organic films, and may also provide some very useful insights into how to construct DNA sensors utilizing a DNA-induced hybridization change in the structure of the alkanethiolate linking layer.

## 2. Experimental section

### 2.1. Materials

Sodium acetate (NaAc; p.a., POCH, Poland), magnesium acetate (Mg(Ac)_2_; p.a., POCH, Poland), potassium persulfate (VWR Chemicals), potassium hydroxide (POCH, Poland), trisodium phosphate (Chempur, Poland), 1× PBS buffer (pH 7.4; Sigma), absolute ethanol (99.8%; POCH, Poland), EDTA (Sigma), tris–EDTA buffer (TE; Sigma), tris(2-carboxyethyl)phosphine hydrochloride (TCEP; Sigma), 2-amino-2-(hydroxymethyl)propane-1,3-diol (Tris; Sigma), 6-mercaptohexan-1-ol (MCH; Sigma) were all of high purity, and were used as received. For all the experiments, we used distilled and deionized water with a conductivity of 0.056 μS cm^−1^ produced by the Hydrolab system. The following oligonucleotides, purchased from MWG-Operon (Eurofins), were used:

• Capture DNA (5′ → 3′): thiol-C6-GCCTTCACAGGGTCCTTTATGT.

• Complementary target DNA (5′ → 3′): ACATAAAGGACCCTGTGAAGGC.

### 2.2. Preparation of thiolated DNA sequences: breaking of disulfide bond

Because thiols are strong nucleophiles and spontaneously form disulfides in neutral aqueous solutions, the thiolated DNA fragments were synthesized and delivered as a disulfide. Therefore, before the use of capture DNA (containing an alkanethiol moiety) for the formation of a DNA layer, the disulfide bond of the thiol-modified oligonucleotide was reduced using the following procedure: the capture DNA in the disulfide form was dissolved in 200 μL of a 10 mM TCEP solution in TE buffer (10 mM Tris, 1 mM EDTA, pH 8.0). The resulting solution was then mixed in an Eppendorf ThermoMixer for 60 min at room temperature. Next, to the mixture obtained, 150 μL of a 0.3 M NaAc and 1 mM Mg(Ac)_2_ solution was added. The tube was filled with absolute ethanol (99.8%), gently shaken, and incubated for 20 min at −20 °C. To isolate the obtained precipitate (HS-DNA) from the solution, it was centrifuged at 13 000 rpm for 5 min. At the end, the pellet was dried at room temperature.

### 2.3. Formation of SERS substrates

Nanostructured hetero-epitaxial GaN samples covered by some plasmonic metals or their alloys were used as SERS substrates. Samples of Si-doped n-type GaN on sapphire, with a free carrier concentration of *n* = 1 × 10^18^ cm^−3^, were photoetched in a 0.02 M K_2_S_2_O_8_ + 0.02 M KOH + 0.02 M Na_3_PO_4_ solution under illumination using a 300 W UV-enhanced Xe lamp (Oriel).^22^ Please note that the standard KSO-D solution (0.02 M K_2_S_2_O_8_ + 0.02 M KOH) used for photoetching during the preparation of previous SERS substrates^19,23^ was modified by the addition of a third component (Na_3_PO_4_) in order to increase the stability of the solution.^24^ On those nanostructured GaN substrates, thin layers of plasmonic metals (Au, Ag, Cu, and Au/Ag alloy – 70/30 wt%) were sputtered. After the silver sputtering, the substrates were packed in a vacuum and unpacked only before the formation of the DNA layers and SERS measurements. This procedure decreases the contamination of SERS substrates and allows them to be stored for longer periods of time.

### 2.4. Modification of the SERS substrates with DNA fragments

The efficiency of the immobilization step of the capture HS-ssDNA as well as the hybridization process requires the presence of the single ssDNA fragments in the solution. For this purpose the solutions of: (i) capture ssDNA (HS-ssDNA) and target ssDNA were heated for 10 min at their melting temperatures (specified by the manufacturer). After that time the DNA solutions were immediately cooled down in an ice bath. To modify the SERS substrate with DNA fragments with alkanethiol moiety, the 30 μL droplet of 2 μM capture HS-ssDNA solution prepared in the ultrapure water was placed on the SERS substrate and left under the cover for 2 h at room temperature. After that time the HS-ssDNA modified SERS substrate was carefully rinsed with ultrapure water to remove any non-specifically attached thiolated capture DNA fragments. Thiolated DNA fragments of substantial length (over a dozen nucleotides) can bind to the gold matrix through thiol groups and/or through nitrogenous bases. To eliminate the possibility of the capture DNA binding to the matrix surface through nitrogenous bases, the sensing layer was sealed with 6-mercaptohexan-1-ol (MCH) by placing 30 μL droplet of 1 mM MCH aqueous solution on the SERS substrate and left for 1 h.^25^ After that time the droplet was carefully rinsed with ultrapure water. The as-prepared sensing layer was ready for the hybridization process. For this purpose the 30 μL droplet of 2 μM solution containing target ssDNA was placed on the SERS-S-ssDNA/MCH substrate and left for 1 h. Next the droplet was carefully rinsed with ultrapure water.

### 2.5. Experimental techniques

Thin layers of various plasmonic metals were sputtered on nanostructured GaN samples using a Quorum Q150TS sputter coater with a function of cleaning oxidized targets.

The surface morphology of the SERS substrates was examined using a Zeiss Ultra Plus scanning electron microscope (SEM).

The Raman measurements were carried out using a Horiba Jobin-Yvon Labram HR800 spectrometer equipped with a 600 groove·mm^−1^ holographic grating, a Peltier cooled charge-coupled device (CCD) detector (1024 × 256 pixels), and an Olympus BX40 microscope with a long-distance 50× objective installed. A diode laser provided the excitation radiation, at a wavelength of 785 nm.

## 3. Results and discussion

### 3.1. SEM characterisation of SERS substrates

**Fig. 2:**
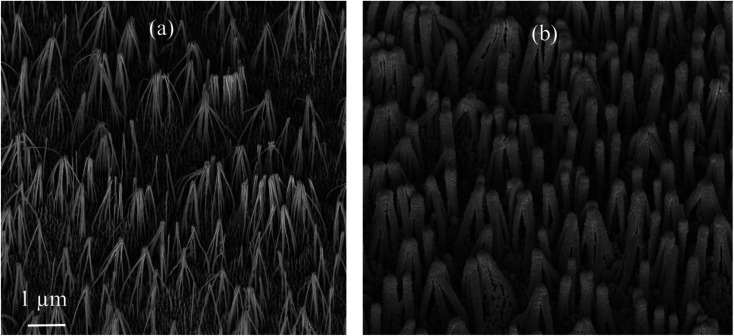
SEM images of a hetero-epitaxial GaN layer after: (a) photoetching and (b) subsequent sputtering of silver. Samples tilted by 45°.

**Fig. 3:**
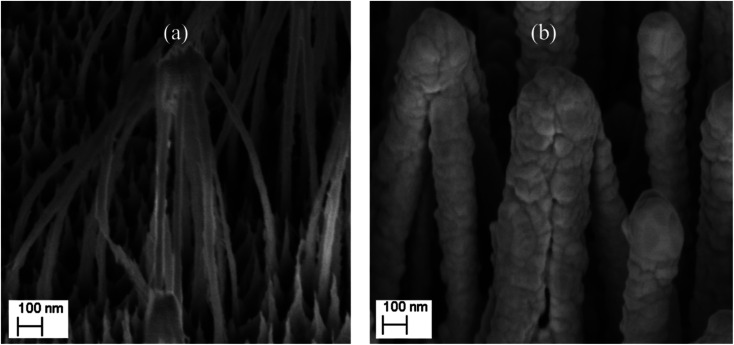
SEM images of a hetero-epitaxial GaN layer after: (a) photoetching and (b) subsequent sputtering of silver. The images were recorded at a larger magnification than those in Fig. 2.

**Fig. 4:**
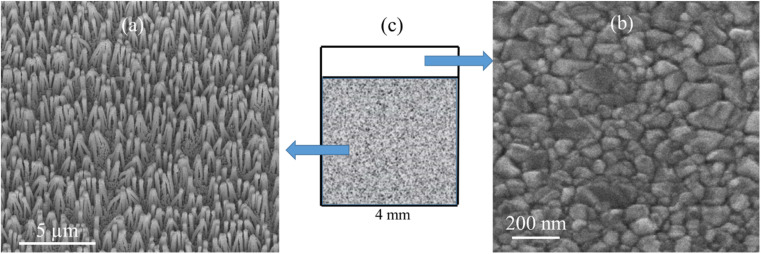
SEM images of: (a) nanostructured and (b) flat part of GaN platforms covered with an Ag layer and used for SERS measurements. (c) Schematic top view of GaN platform. When recording image (a), the sample was tilted by 45°.

SEM images of the nanostructured GaN after photoetching are shown in Fig. 2a and 3a (the images were recorded using different magnifications). The nanopillars shown in Fig. 2a and 3a were formed on dislocations due to the effective recombination of photo-carriers along the linear defects.^22^ On such nanostructured substrates, thin layers of gold, silver, copper and Au/Ag alloy were sputtered. The time of sputtering of all plasmonic metals was constant (250 s). A sample morphology of the substrate after deposition of a silver layer is shown in Fig. 2b and 3b, while images of gold-covered substrate are shown in Fig. S1.† The overall structure of the samples used for SERS measurements was very uniform on the 4 mm × 4 mm surface of the SERS substrates (see Fig. 4a). Apart from the photoetched area, each sample had a flat, not etched 1 mm × 4 mm band (the upper part of the sample shown in Fig. 4c) that was also covered by plasmonic metal. Using high magnification SEM images (see Fig. 3), the thickness of the sputtered metal layer was established to be about 75 nm, which was enough to prevent the appearance of a Raman spectrum from the underlying GaN (*Φ*_GaN_ = 50 nm – see Fig. 3a, *Φ*_GaN+Ag_ = 200 nm – see Fig. 3b, therefore *d*_Ag_ = (200 − 50)/2 = 75 nm). The metal layer formed was not continuous, but consisted of nanoclusters of less than 100 nm size (see Fig. 3b and 4b). It was previously shown that SERS measurements performed on the nanostructured parts of GaN substrates yield up to three orders of magnitude higher SERS enhancement factors than measurements taken on the flat parts.^23^ This effect was attributed to the presence of hot-spots that formed due to the more complex morphology of the photoetched GaN surface.

### 3.2. SERS measurements

**Fig. 5:**
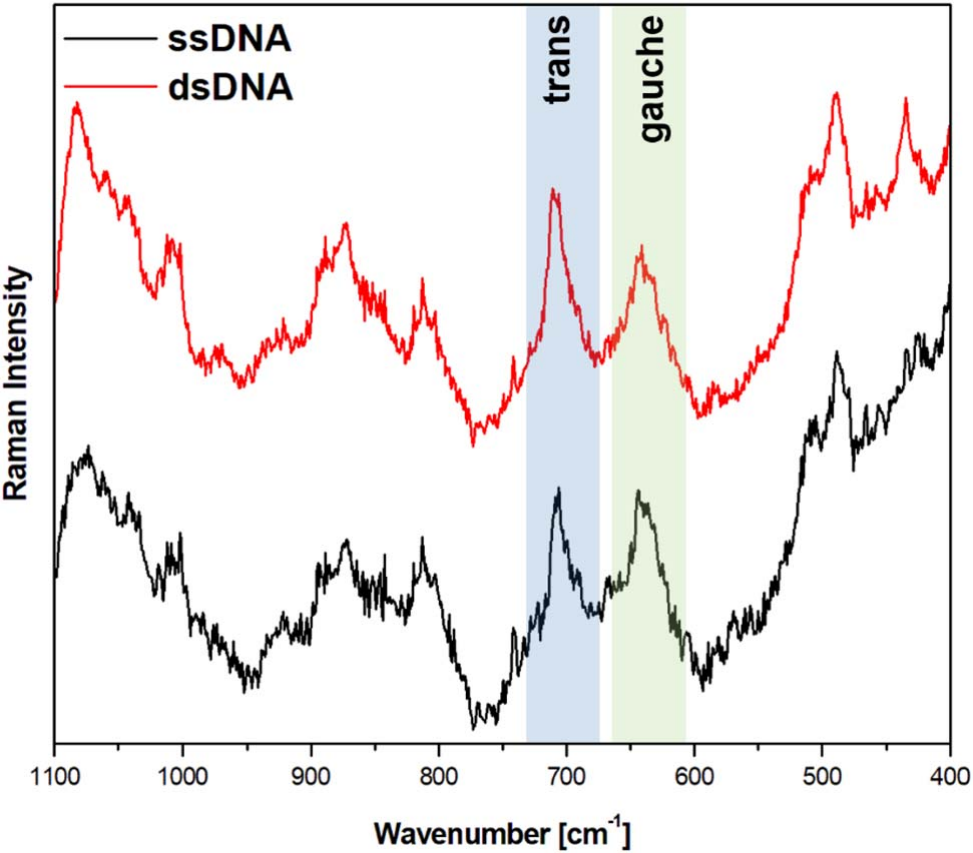
SERS spectra of a gold-covered nanostructured GaN substrate modified with capture HS-ssDNA and MCH before (black line, marked also ssDNA) and after (red line, marked also dsDNA) interaction with the solution containing complementary ssDNA. The spectra presented were averaged from 400 spectra recorded at different places on the SERS substrate.

**Fig. 6:**
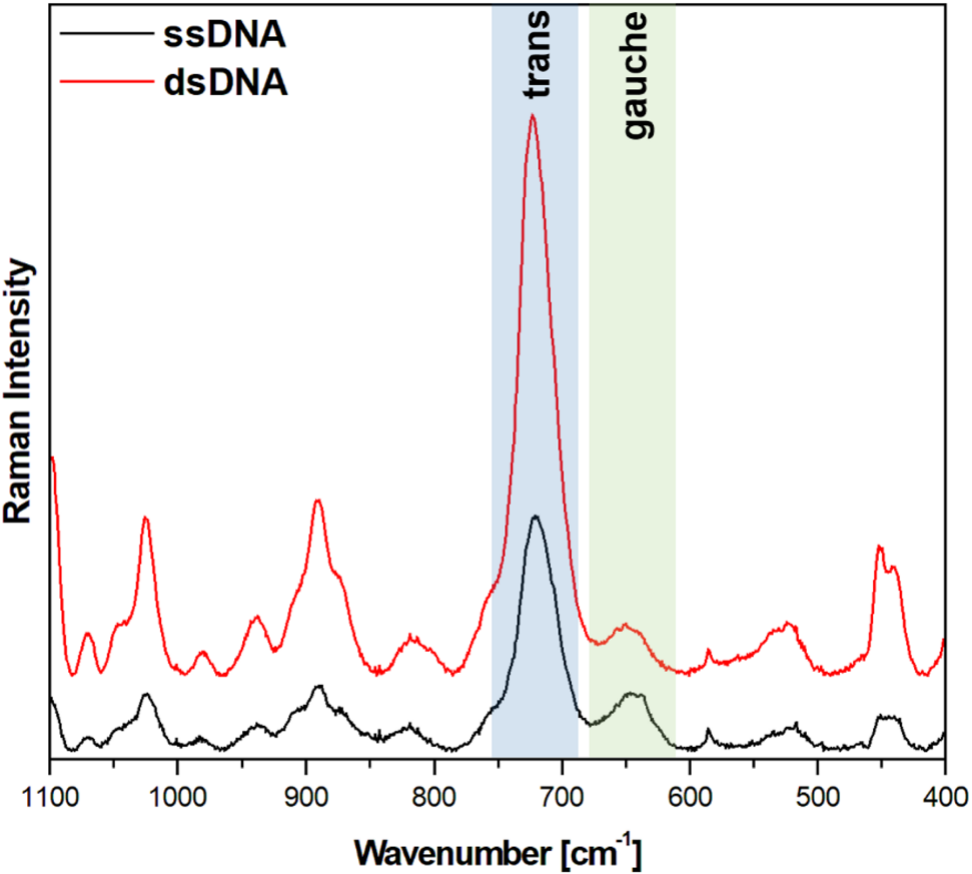
SERS spectra of a silver-covered nanostructured GaN substrate modified with capture HS-ssDNA and MCH before (black line, marked also ssDNA) and after (red line, marked also dsDNA) interaction with the solution containing complementary ssDNA. The spectra presented were averaged from 400 spectra recorded at different places on the SERS substrate.

**Fig. 7:**
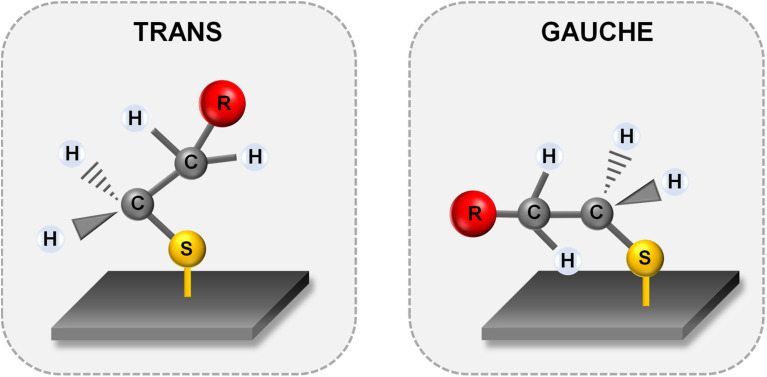
Structures of the Me–S–C–C chain of alkanethiolate chemisorbed on the metal surface in the *trans* and *gauche* conformations. R – substituent, *e.g.* ssDNA, carbon chain.

Fig. 5 and 6 show the average (from 400 individual measurements at various places of the nanostructured SERS substrate) SERS spectra measured on the GaN substrates covered with a layer of gold or silver and modified with DNA and MCH. The spectra presented were measured before and after the hybridization of the immobilized thiolated capture HS-ssDNA with the fully complementary target ssDNA. The most characteristic Raman bands, which appear in the presented wavenumber region and which are marked in Fig. 5 and 6, are the bands near 700 cm^−1^ (the pair of bands at 641 cm^−1^ and 711 cm^−1^, in the case of gold, and at 646 cm^−1^ and 721 cm^−1^, in the case of silver). These bands are due to the *ν*(C–S) stretching vibration of the alkanethiol moieties adsorbed on the metal surfaces. The *ν*(C–S) band at a lower wavenumber (641/646 cm^−1^, for the measurements on Au and Ag, respectively) is characteristic for an alkanethiol moiety having the *gauche* conformation of the Me–S–C–C chain (see Fig. 7), whereas the *ν*(C–S) band at a higher wavenumber (711/721 cm^−1^, for measurements on Au and Ag, respectively) is characteristic for an alkanethiol moiety having the *trans* conformation of the Me–S–C–C chain (see Fig. 7).^26–29^ Both the *gauche* and *trans ν*(C–S) bands are shifted towards lower wavenumbers in comparison with the respective bands observed in the Raman spectrum of liquid 6-mercaptohexan-1-ol (at 656 and *ca.* 740 cm^−1^ – Fig. S2†). This shift was related to a withdrawal of electron density from the C–S bond due to the bonding of the sulphur atom with the metal surface.^26,30^ It is worth mentioning that, although the alkanethiolate moiety is only a very small part of the DNA + MCH layer formed on the SERS-active substrate, due to the very surface-sensitive mechanism of SERS enhancement (as mentioned in the introduction, the SERS signal strongly decreases with increasing distance from the plasmonic substrate – roughly as a function of *r*^−10^),^10–12^ the bands from the vibrations localized in those parts of the layer that directly interact with the metal substrate (as the Me–S–C–C chain) dominate the resulting SERS spectrum.

As can be seen from a comparison of the SERS spectra presented in Fig. 5 and 6, the signal-to-noise ratio is significantly larger in the SERS spectra recorded from the GaN substrate covered with a silver layer than with a gold layer, although both measurements were made under the same experimental conditions (thickness of the deposited metal layer, power of the laser beam, accumulation time, objective installed in the confocal microscope, *etc.*). Also, the hybridization with complementary ssDNA induced a significantly larger change in the ratio of the average intensity of the *trans* and *gauche ν*(C–S) bands in the experiments on silver – from 4.1 to 10.8 (a change of 263%), than in those on gold – from 0.99 to 1.28 (a change of 29%). This means that silver substrates are significantly more promising for the SERS observation of the rearrangement of the chains of chemisorbed ω-substituted alkanetiols (MCH and the alkanethiol linking moiety of HS-ssDNA) induced by complementary DNA than the previously used gold substrates.^19^

**Fig. 8:**
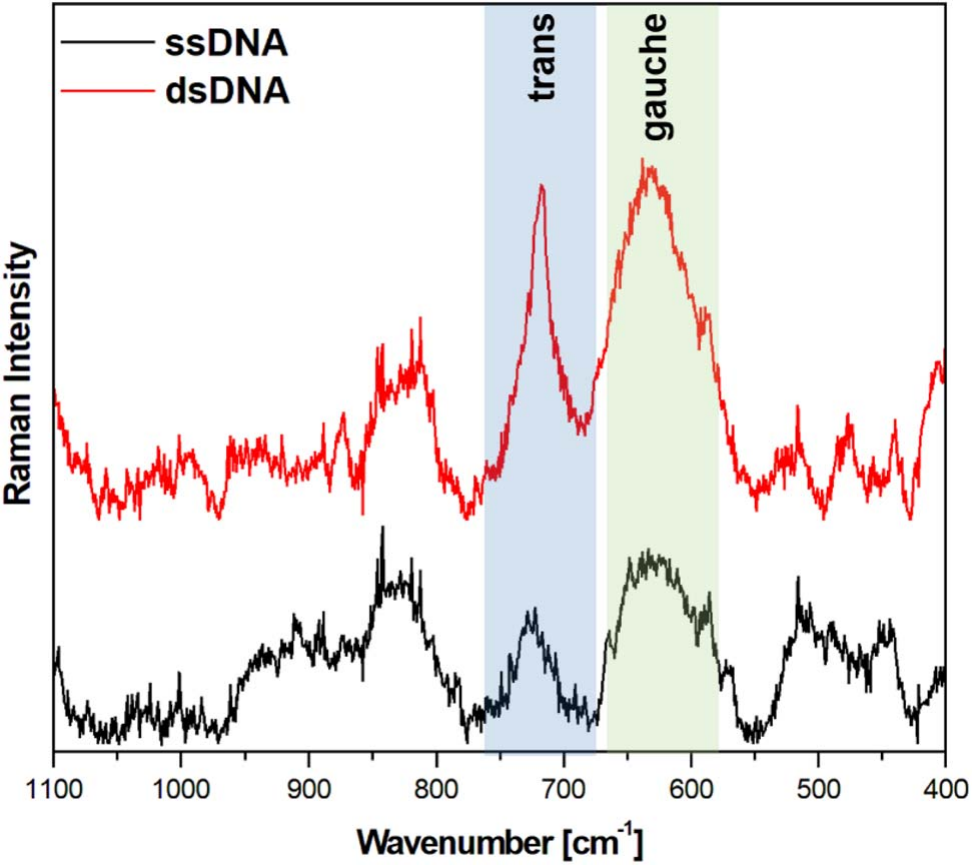
SERS spectra of a nanostructured GaN substrate covered with an AuAg alloy (with an atomic ratio of Au : Ag = 57 : 43) and modified with capture HS-ssDNA and MCH before (black line, marked also ssDNA) and after (red line, marked also dsDNA) interaction with the solution containing complementary ssDNA. The spectra presented were averaged from 400 spectra recorded at different places on the SERS substrate.

Because gold and silver easily form an alloy, we also decided to carry out an analogous comparative analysis using a SERS substrate covered with an Au : Ag alloy, where the atomic composition of Au and Ag was equal to 57 : 43 (70 : 30 wt%). The results obtained in the measurements made using this alloy were similar (in terms of both the ratio of the intensity of the *trans* and *gauche ν*(C–S) bands and the noise level) to those obtained on pure gold substrates – see Fig. 8.

In addition to gold and silver, which are standardly used to carry out SERS measurements, large SERS enhancement factors are also generated on copper substrates,^31,32^ and copper seems to be the third most frequently used metal in SERS measurements. Therefore, we decided to carry out similar experiments using copper layers deposited on nanostructured GaN. Unfortunately, in such systems, we have not obtained good quality and reproducible SERS spectra, which means that copper substrates are not very useful for such SERS measurements.

As can be seen from the SEM images of the deposited metal films (Fig. 4b), these layers revealed clearly visible nanostructuring (they were composed from densely packed metal nanoclusters several dozen nanometers in size) even when the plasmonic films were sputtered on a flat GaN surface. Layers of densely packed nanoclusters of plasmonic metals are often highly SERS-active, so we decided to compare the intensity of the SERS spectra of DNA films formed on silver layers sputtered on flat and nanostructured GaN surfaces. We found that the intensity of the SERS spectra measured on silver layers sputtered on photoetched GaN surfaces were at least 2 orders of magnitude larger than the intensity of the SERS spectra of analogous structures, but sputtered on the flat parts of the GaN substrates. This means that the nanostructuring of GaN substrates by photoetching has a very large effect on the SERS activity of the nanomaterial formed, a result that remains in agreement with previous reports.^23^

## 4. Conclusions

6-Mercaptohexan-1-ol and capture single-stranded DNA with an attached alkanethiol linking moiety were chemisorbed on surfaces of nanostructured GaN covered with sputtered layers of certain plasmonic metals. Hybridization with the target ssDNA, complementary to the chains of immobilized thiolated capture HS-ssDNA, induced changes in the conformation of the alkanethiol chains of blocking and linking moieties. We found that such changes are significantly larger in the case of experiments on silver than on gold: when the measurements are carried out on a silver layer the change in the ratio of intensities of the *trans* and *gauche ν*(C–S) bands was approx. 260%, whereas in the case of experiments on a gold the change in the ratio of the intensities of the *trans* and *gauche ν*(C–S) bands was only about 30%. Moreover, the intensity of the SERS signals in the measurements on silver was significantly larger than those observed on other SERS substrates. This means that silver is significantly more promising for DNA SERS sensors utilizing such hybridization-induced rearrangement than the gold SERS substrates previously used.

## Author contributions

Aleksandra Michałowska contributed to conceptualization, investigation, collection of data, data curation, designing of some figures, writing the original article and its reviewing. Aleksandra Gajda contributed to investigation. Agata Kowalczyk, Jan L. Weyher and Anna M. Nowicka contributed to conceptualization, investigation, writing the original article and its reviewing. Andrzej Kudelski contributed to conceptualization, writing the original article and its reviewing, supervision and funding acquisition.

## Conflicts of interest

There are no conflicts to declare.

## Supplementary Material

RA-012-D2RA05318G-s001
