# Supplementary material for: Surface-enhanced Raman scattering used to study the structure of layers formed on metal surfaces from single-stranded DNA and 6-mercaptohexan-1-ol: influence of hybridization with the complementary DNA and influence of the metal substrate
Source: RSC Adv. 2022 Dec 8;12(54):35192–8. doi: 10.1039/d2ra05318g (PMC9730742; doi:10.1039/d2ra05318g)
Supplement: RA-012-D2RA05318G-s001 [file RA-012-D2RA05318G-s001.pdf]

Electronic Supplementary Material (ESI) for RSC Advances.
This journal is © The Royal Society of Chemistry 2022

Supplementary information

Surface-enhanced Raman scattering used to study the structure of layers formed on metal surfaces from single-stranded DNA and 6–mercaptohexan–1–ol: Influence of hybridization with the complementary DNA and influence of the metal substrate

Aleksandra Michałowska[1], Aleksandra Gajda[1], Agata Kowalczyk[1], Jan L. Weyher[2], Anna M. Nowicka[1], Andrzej Kudelski[1*]

[1] Faculty of Chemistry, University of Warsaw, Pasteura 1 Str., PL 02-093 Warsaw, Poland

[2] Institute of High Pressure Physics of the Polish Academy of Science, Sokolowska 29/37 Str., PL 01-142 Warsaw, Poland

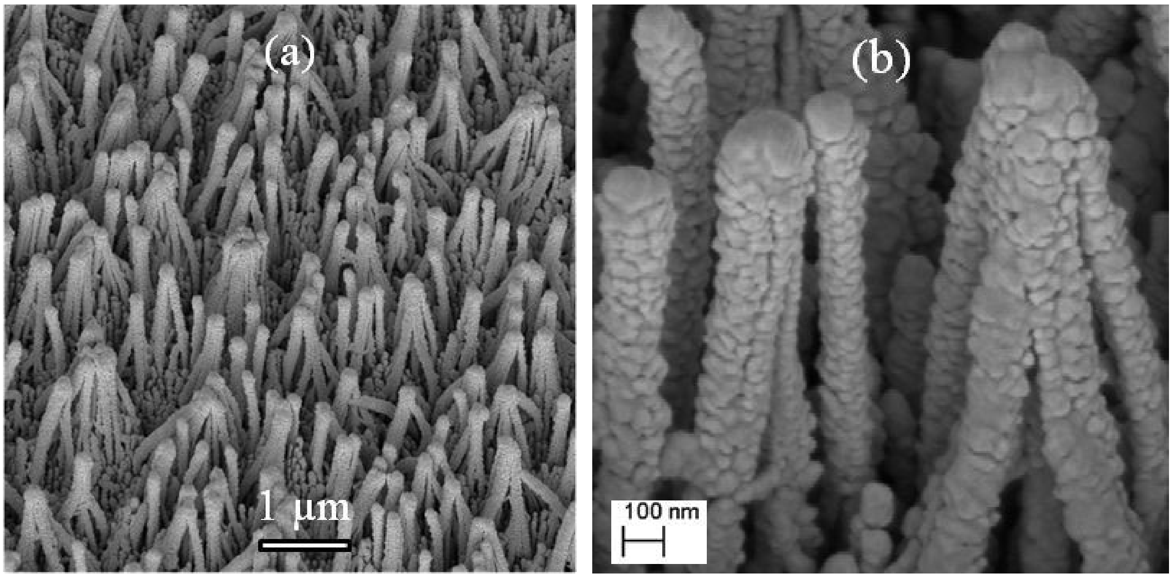

Figure S1. SEM images of a hetero-epitaxial GaN layer covered by gold.

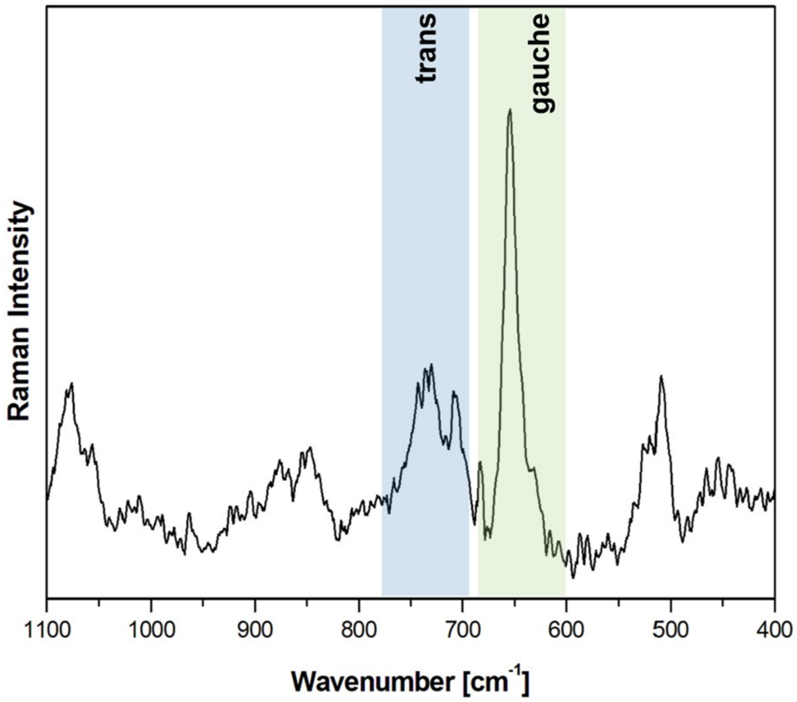

Figure S2. Raman spectrum of liquid 6–mercaptohexan–1–ol.
